# Clinical educator's experiences of the decentralised training platform for Occupational Therapy students in KwaZulu-Natal, South Africa

**DOI:** 10.4314/ahs.v21i4.53

**Published:** 2021-12

**Authors:** Belinda M Malinga, Deshini Naidoo, Thavanesi Gurayah, Pragashnie Govender

**Affiliations:** Discipline of Occupational Therapy, School of Health Sciences University of KwaZulu-Natal, Westville Campus, Durban, South Africa

**Keywords:** Decentralised training, clinical educators, service learning

## Abstract

**Background:**

In piloting a shift from traditional practice-based placements to decentralised clinical training (DCT), there was a need to explore the factors that influenced the placement as part of monitoring and evaluation. DCT involves placement to clinical sites away from the higher education institution necessitating changes to supervision strategies utilised.

**Objective:**

This study explored the experiences of clinical educators supervising occupational therapy students within this new model during a pilot phase of the DCT programme at one institution in South Africa.

**Method:**

The study was located in KwaZulu-Natal province and followed an explorative qualitative design with semi-structured interviews and focus groups with purposively sampled clinical educators (n=11). Data were audio-recorded and d thematically analysed.

**Findings:**

Two central themes emerged and included the clinical educators' expectations (organisation factors, role and scope of partners in decentralised training and communication) and experiences (perspectives and value of decentralised training).

**Conclusion:**

Decentralised training has considerable potential to contribute to authentic student learning. Improved communication between all stakeholders would assist in enhancing the quality of the learning experiences on such platforms. Students need to be more prepared prior to commencing DCT, and there is a need for more rural placements with a primary health care focus.

## Introduction

The burgeoning number of students within health professions education has necessitated the acquisition of additional practice-based placement (PBP) sites and the adoption of alternative modes of student supervision. Decentralised clinical training (DCT) is a model of supervision and a platform for PBP. Practice-based placement refers to students being allocated to a clinical or non-governmental organisation (NGO) site to develop professional reasoning, practice skills and behaviours.^1^ Practice-based placements form the foundation that facilitates students' acquisition of profession-specific clinical reasoning and practice.^1^,^2^ Traditional PBP comprised of approximately five students placed at a hospital, NGO, or school geographical area, around the higher education institution (HEI) for a six-week duration. Supervision of traditional PBP for final year students in occupational therapy at the HEI consisted of approximately two hours of contact time per week at the PBP site; with the academic supervisor responsible for assessing intervention sessions. DCT differs from traditional PBP as students are trained away from the central academic hospitals, at district or regional level hospitals or at appropriate healthcare facilities, which provide services to the surrounding communities. The occupational therapists at the PBP site supervise daily professional practice while the academic supervisors provide distant supervision using digital platforms such as Zoom, Skype or WhatsApp calls (including videocalls) due to the geographical distance of the sites^2^. Both traditional and DCT PBP meet the criteria for the 1000 clinical hours required by the international and local regulatory bodies, namely, the World Federation of Occupational Therapy (WFOT) and the Health Professions Council of South Africa (HPCSA).

There are numerous benefits to DCT that are acknowledged in the literature.^2^–^7^ During PBP on the DCT platform, students are exposed to the practical realities of working in the resource-constrained public health sector, and have increased opportunities for independent professional practice.^2^,^3^,^4^ Additionally, during DCT students gain experience in adapting their professional practice interventions to accommodate the service users' social determinants of health. They also tend to acquire an understanding of the public health system and the role of context in health and illness^3^,^4^,^5^,^6^. Practice-based placement on DCT platforms aim to improve students' attitudes toward rural healthcare, work in the public sector, and in engagement and appropriate responses to the communities they are embedded within.^4^,^5^,^6^,^7^ Students would be more adequately prepared for their first year of work (community service) and future professional practice through this exposure to the public sector and rural practice realities.^4^,^7^ The training of these healthcare professionals, using the DCT model of PBP are underpinned by social accountability principles.^8^ As described by the World Health Organisation (WHO), the responsibility is to align the students' education and professional actions, with the primary health issues of the communities served.^8^

A key driver prompting the shift from traditional PBP sites to DCT is to improve graduates' preparedness to cope with service-delivery during community service. Community service practitioner's difficulties with adjusting to the reality of service delivery, and the diversity of their placements, has been acknowledged in the literature.^2^,^4^,^8^,^9^,^10^ In a study by Beyers, community service practitioners who were deployed at rural health facilities, were primarily embedded in the process of adapting to a new environment and professional expectations, rather than focussing on service delivery.^11^ Challenges such as feeling isolated due to a lack of support and being away from home, as well as being unequipped for professional practice whilst assuming increased professional accountability were also noted in other studies.^2^,^9^,^10^,^13^ Similarly, Naidoo and colleagues^14^ found that final year occupational therapy (OT) students were only partially prepared for their community service year of practice. This, was attributed to their lack of confidence in applying newly developed professional skills and perceived inability to apply the OT process adequately.^14^

The KwaZulu-Natal (KZN) Department of Health (DoH) sought to address community service practitioners' level of preparedness by engaging in a memorandum of understanding with the HEI in this study^15^. One of the fundamental tenets of this agreement was the need to decentralise the PBP to support primary healthcare (PHC) re-engineering. In this process a smoother transition between health professionals' training and their ability to practice more autonomously on the service delivery platforms was envisaged.^15^ Decentralised training was first piloted at the HEI in the College of Health Sciences (CHS) in 2017.^2^ The OT programme at the HEI implemented DCT in 2018. The OT programme is a four-year degree, with only final year students placed on the DCT platform. For DCT PBP, final year students are placed throughout KZN (from UMgungundlovu to Manguzi), in public sector healthcare facilities for a period of up to six weeks, for two blocks (physical and psychiatric fieldwork blocks). The hours accumulated contribute toward the students acquisition of the require1000 clinical hours required by WFOT and HPCSA. The choice of placement site is dependent on several factors. These include the willingness of the site to accept the student, the presence of a registered OT at the site, and the willingness of the HEI in providing accommodation for students in close proximity to the site. In the pilot, students were placed in tertiary, specialised, and district hospitals located in urban and rural areas, geographically away from the HEI.

Academic supervisors provided distance supervision via weekly meetings (either telephonically or via digital platforms) while clinical educators offered daily supervision. Each DCT PBP received a minimum of two to a maximum of four final year OT students. Clinical educators were expected to provide daily supervision of professional practice and assess students' professional practice performance during the placement using a university designed template. Clinical educators were also expected to assess an intervention demonstration toward the end of the placement. The professional practice performance mark and the intervention demonstration mark contributed toward the semester mark of the module. For the context of this study, the OT providing supervision at the facilities are referred to as clinical educators, and the academic staff representing the university are the academic supervisors.

Miles and colleagues conducted a study at UKZN that explored students' and academics' perspectives of their experiences of DCT.^16^ This study is, therefore, suitably positioned to explore clinical educators' perspectives to present a holistic picture of the pilot roll-out of PBP within DCT in KZN.

## Literature review

Decentralised training plays a role in addressing the inequality in service delivery to public sector facilities, including rural health facilities.^3^,^4^,^5^,^6^,^7^ It aims to provide future healthcare professionals (HCPs) with an opportunity to utilise and practice essential competencies required for community engagement and to ensure quality healthcare services,^10^,^12^,^13^,^17^ which is operationalised more autonomously for the first time during community service.

As it is more commonly known, the internship and community service programme, or community service, is a one-year requirement fulfilled by most HCPs after the accomplishment of their undergraduate degrees.^17^ Community service aims to improve access to health resources, particularly in rural communities and facilities, through the HCP placement in rural and poorly staffed public sector facilities.^19^ Paliadelis^19^ defines rural contexts as areas with smaller populations, a distance from major cities, and those that generally have a ‘corresponding lack of access to the full range of services and infrastructure.’^19^,^2^ Healthcare professionals display a preference for servicing well-resourced urban healthcare facilities, and therein lies inequitable services in the public sector, including rural healthcare facilities.

The National Health Insurance (NHI) is the financing vehicle through which the national DoH plans to deliver universal health coverage.^20^ The re-engineered PHC approach has also shifted the focus of intervention from using a purely curative approach, to including promotive, preventative and rehabilitative approaches.^16^ The UKZN CHS, in collaboration with KZN DoH, embarked on the roll-out of DCT to equip health science students with the necessary graduate attributes and competencies to serve the communities in which they are placed.^2^,^16^ Still, DCT is relatively new within the CHS; therefore it is vital to monitor the factors that promote and hinder learning and to adapt the process of DCT timeously to ensure a positive learning experience.^2^ The Integrated DCT (i-DeCT) project was introduced to respond to this need by exploring factors that would influence DCT placements' roll-out within the CHS at the HEI. This was aimed towards developing a model of practice for service learning that would be most suitable within the CHS.^2^

A perusal of the literature assisted in a deeper exploration of the application of DCT. De Villiers et al^21^ scoping review highlighted that the literature on DCT could be categorised into four themes, namely student learning, the training environment which refers to the context of the DCT sites, including the clinical educators, community role, as well as leadership and governance.^21^ Student learning included the various student experiences of DCT, including curriculum renewal as part of the DCT process.^21^ Electronic communication and internet access were reported to be important in relieving isolation at DCT sites.^21^ The role of the community highlighted student engagement at the community level, while leadership and governance highlighted stakeholder engagement as well as visionary leadership and funding.^21^

De Villiers et al^21^ further highlighted that clinical educators described greater job satisfaction, workforce retention, professional development and a positive impact on students, as benefits of DCT. Similarly, Mlambo and colleagues' scoping review on DCT considered four themes namely rural workforce training, development of context-specific competencies for raising social accountability, support for community-based clinical teaching (CBCT), and community engagement.^3^ Both De Villiers et al^21^ and Mlambo et al^3^ reviews highlight the importance of community-level engagement with the DCT platform stakeholders. In their review, Mlambo et al highlighted CBCT as improving students' knowledge of rural healthcare and in fostering an overall positive attitude towards DCT^3^. Community-based clinical teaching benefited the student and community engagement by the provision of PHC services that are ‘community-oriented’, as well as improving student familiarity with the healthcare system.^3^ There is also evidence to indicate that with the introduction of DCT and various other programmes, students who were allowed to practice in rural or underserviced areas as part of their undergraduate studies, were more likely to be inclined to practice in these areas once they qualified.^4^,^5^,^6^,^7^ It is essential to note that the OT programme at the HEI placed students in predominantly tertiary and regional hospitals in urban and peri-urban sites that were geographically away from the university which differed from the definition of DCT in most literature, which emphasises rural placement and rural health.

This study aimed to explore the experiences of clinical educators in students' supervision to gain insight into their experiences during the pilot phase of the DCT implementation. The study further aimed to gain insight to the factors that need consideration for a positive learning experience for OT students on DCT platforms.

## Methods

An explorative qualitative research design was used to explore the lived experiences of OT clinical educators involved in the roll-out of DCT in KZN.^22^,^23^ The research was conducted in selected healthcare facilities in three districts, namely eThekwini, uMgungundlovu and Zululand of KZN province in South Africa. Eleven participants from these districts were recruited using purposive sampling.^23^ The selection of the participants were based on specific inclusion criteria. Participants had to be registered with the HPCSA, employed in the relevant DCT healthcare facilities at the time of data collection, and provided supervision to OT students from the HEI for a minimum of six weeks, for the 2018/2019 pilot year of DCT.

A pilot study was conducted prior to initiation of the study allowing for refinement of interview questions. This included a pilot triad interview with three participants that were exposed to the supervision of OT students. In the main study, semi-structured individual interviews (n=2), two dyad interviews (n=4) and one focus group (n=5) was used to collect data to gain insight into the participants' perspectives on DCT. All interviews were conducted in English and were approximately 45 minutes to an hour in duration. The interviews were audio-recorded, and data were transcribed verbatim. Data were analysed thematically using codes, categories, sub-themes and themes.^24^

The trustworthiness of the study was confirmed by ensuring credibility, dependability and confirmability.^25^ The credibility of the study occurred through the use of purposive sampling to ensure that all the participants who had a rich knowledge of training OT students on the DCT platform were included in the study. Dependability in the research study was ensured through the use of a consistent interview schedule. There were additional probe questions that allowed deeper probing into specific issues, whilst using a consistent schedule of questions, allowed the research to be replicated. Confirmability was ensured through collecting data until redundancy was reached, and which allowed for multiple viewpoints on the topic.^25^ The first author, who had no involvement in DCT supervision, collected the data and all the authors were involved in data analysis. This reduced potential researcher bias.

Ethical approval was obtained from a Humanities and Social Sciences Research Ethics Committee (HSS/0727/017) at the HEI prior to study commencement. Gatekeeper permission from the Health Research and Knowledge Management Directorate of the Provincial DoH was obtained prior to accessing the participants at the various DCT sites (NHRD ref KZ_201805_007). Informed consent was obtained from all participants prior to the commencement of the study and participants were informed that they were allowed to withdraw from the study at any time and that their participation was voluntary.

## Results

A total of 11 participants out of a possible 16 participants were included in the study. Table 1 illustrates the characteristics of clinical placement sites. The sites included varying levels of care, including specialised psychiatric sites, that were willing to accept students for a practice-based placement.

**Table 1:** Characteristics of Clinical Placement Sites

	*Clinical Block*	*Acute or Chronic*	*Facility Type*	*Area*
**SITE A**	Psychiatric	Acute	Tertiary Hospital	Urban
**SITE B**	Physical	Acute	Regional Hospital	Semi-rural
**SITE C**	Physical	Acute	Tertiary Hospital	Urban
**SITE D**	Psychiatric	Acute and Chronic	Specialised	Urban
**SITE E**	Psychiatric	Acute	Specialised	Urban

The participants of this study were predominantly female (82%; n=9). There was a degree of diversity with the sample involving African (27%; n=3), Asian (36%; n=4) and Caucasian participants (36%; n=4). More than half of the participants (64%; n=7) had less than five years of experience in supervising students. The majority (55%; n=6) of the participants achieved their qualification at the UKZN.

The findings were categorised into two main themes, namely: the clinician's expectations versus reality after DCT implementation and the clinician's perspectives on DCT.

## Theme 1: Clinical Educators Expectations of DCT vs Reality

### Organisational Factors

All the participants reported time as a barrier during DCT. The participants reported that they underestimated the amount of work associated with providing supervision to the students. As a result, they had to allocate a significant amount of time to the students, which inevitably affected their clinical work. Difficulties with managing their own time between providing supervision to the students and administrative tasks required of DCT and their clinical workloads within their facilities were cited.

“... *it was quite challenging for us in that we had to manage our time to watch sessions, and mark write ups, and give feedback*.” (Semi-structured group, Participant 8)

All the participants emphasised that they had envisioned having more physical support from the academic supervisors. However, they reported that the academic supervisors did not visit their respective facilities to assist with student supervision, observation and feedback. There was consensus amongst the participants that they required more ‘physical presence’ from the academic supervisors.

“*[I] think general problems we had, the academic supervisors not coming to the institution, even to examine one student in a session and understanding the context. I think that was our biggest problem we faced, we had to ask the academic supervisors to now come to us to get to know the institution, but we didn't get that support of them coming and observing the sessions with students.*” (Semi structured group, Participant 10)

Prior to the commencement of DCT, each of the participants had their own expectations of the students and their preparedness for DCT. There was unanimity amongst the participants that they had expected the students to be sufficiently prepared to cope with service delivery within the health facility. The participants had anticipated the OT students to be more independent, given that they were in their final year of study. The students' level of preparedness, however differed from the participants' expectations. This leads to questions regarding the students' academic readiness, as the participants needed to teach students aspects of professional practice that they felt the students should have already acquired, as vocalised by participants.

“*We really felt that there should have been a higher level of independence*.” (semi-structured group, Participant 1)

“*I think that if you expecting 4th year students to come to your hospital, you expecting them to be independent and competent in their assessment. But, in reality they weren't really ready... so it required more hands on with the students than it should have*.” (semi-structured group, Participant 8)

The logistics of DCT proved to be a challenge. All participants had anticipated that students would have punctual transport and come to the placement site with adequate resources. However, most participants reported experiencing challenges with student transport, which often resulted in the late arrival of students to the sites. Some of the participants reported that they transported students to and from the facilities themselves on occasion to counteract the effects of the delays caused by transport issues. The lack of electronic and therapeutic resources was cited as an additional barrier to effective learning for students. These resources included printers, photocopiers, internet access and Wi-Fi, and therapy equipment. While one participant reported that they did not have any challenges with therapy resources, as they were a well-resourced facility, most of the participants reported that having students placed a strain on their electronic resources, such as computers, photocopiers, and projectors as illustrated by the verbatim quotes.

We didn't have problems in terms of space, however resources were a bit of a problem... computers and photocopying machines, projectors, etc., because we had to use our phones to do the calls and other things.” (semi structured group, Participant 9) “*In terms of resources, this a resource deficient facility... amongst us there are 2 computers for 5 people*..” (semi-structured group, Participant 10)

### Role and Scope of Partners in DCT

The majority of the participants shared similar views on their role as providing supervision to students and guiding them through intervention. There appeared to be confusion between the participants' perceptions of their roles and those of the academic supervisor within DCT. The participants expressed that they felt that their role as clinical educators should not include teaching students what they should already have known, nor should they be involved in assessing students and determining their level of competency. “*We should only be doing the supervision of students, and that was the role of clinicians, not to assess them, or determine their level of competency*.” (semi-structured group, Participant 5)

### Communication

The participants had anticipated clear communication between the placement site and the academic supervisors. Most participants were ambivalent regarding the electronic, written and physical communication between the clinical educators and the academic supervisors. Whilst some participants reported good ongoing communication, other participants felt that the communication was inadequate.

“*...we had telephonic and video conferencing with them [academic supervisors]. That was good, we liked the video conferencing with them because it gave us the opportunity to discuss... and we had constant email with them*.” (Semi-structured group, Participant 5)

“*They could have tried to at least communicate with us, they could have tried to email us. The only call I got at any point this year from any supervisor was when there was a student who was sick...*” (Semi structured group, Participant 1)

## Theme 2: Clinical Educators Perspectives on DCT

### Diverse Experiences

The participants had diverse experiences of DCT. The majority of the participants reported that they experienced feelings of anxiety due to the uncertainty of expectations from themselves and the students, whilst other participants reported that they were enthusiastic and ready to share their knowledge.

“*...I think initially we were quite anxious... like it was the first time we had DCT students. It was the unexpected, not really being sure of what the expectations were.*” (Semi-structured focus group, Participant 4) “*...Well when I heard about it, I was excited and worried at the same time, because remember we attended meetings and when they were explaining how it was going to go about obviously we were sceptical about how successful it was going to be, at the same time I was happy and ready to share my knowledge with students*.” (Semi structured interview, Participant 3)

### Value of DCT

The perspectives of the participants on the value of DCT differed. One of the participants reported that she felt that DCT was unrealistic at their facility. It is a well-resourced site, which did not provide the students with a realistic experience of working at a district-level hospital. Half of the participants reported that there was potential in DCT, and that it was a “work-in-progress”.

“*I don't see it as valuable yet. I see it as a work in progress. I feel they (students) need to do a lot more*.” (semi structured group, Participant 1)

The majority of participants reported that accommodating students for DCT was beneficial for them (participants) and clients. Some of the participants reported that because of the staff shortages, having the students in their respective facilities improved human resources, which meant that more clients could have access to therapy. Their overall statistics improved in those periods. However, one participant mentioned that even though the facility statistics did improve, she did not feel that the quality of work improved.

“*Our stats they really got better, they contributed well and seeing our patients... for some students there was positive feedback from the patients, that they actually contributed therapeutically in their lives*.” (Semi structured group, Participant 9) “*We had extra resources, like the students ran groups uh, educational groups*” (Semi structured group, Participant 6) “Yeah, groups that we wouldn't necessarily have done (Semi structured group, Participant 8)

Some participants mentioned that the value of the service-learning experience on DCT was dependent on the placement site for example, having resources such as splinting pans etc. was not a true reflection of the conditions the students would experience during community service. The majority of the participants reported that even though the students came in with limited preparation, it was a positive experience witnessing their own growth through their service-learning block and the student's personal growth. All of the psychiatric facilities reported that they were pleased that student perspectives on psychiatric OT had changed in addition to student growth.

“*I think being placed at a tertiary hospital, it's a bit unrealistic because at community service you won't get a splinting pan or splinting material, a pressure garment, not even a sewing machine. So I think that maybe the hospitals would have been beneficial to the students, so* yeah*... it definitely depends on where they are sent*.” (semi structured group, Participant 8) “*Students said they were never excited by psych, but now they are excited for it, so it's rewarding as psych is undervalued as an area of expertise*.” (semi structured group, Participant 10)

## Discussion

In this study, the experiences of clinical educators involved with OT students in a DCT platform was explored. A few key issues arose and are discussed within the context of available literature.

A significant issue raised was around the expectations and preparedness of students. Clinical educators expected students to be more autonomous, especially taking into account that they were going into their final year of study; a concern well documented in the available literature on DCT.^4^,^12^,^16^ In this study, however, it emerged that students required more assistance than the clinical educators had anticipated. The implication is that PBP on a DCT platform requires careful planning and timing, to ensure that students are adequately prepared.^13^,^16^,^19^ This view is supported by a recent physiotherapy study on DCT at a HEI in KZN, which suggested that student preparedness needed to be interrogated.^9^,^16^ Moreover, the authors asserted that the gaps around the development of core competencies and social responsiveness of students in undergraduate programmes needed to be addressed.^9^,^11^,^16^

Some measures that were in place included the following. As part of the introduction to DCT, the co-ordinators of the programme developed an orientation that was to be implemented prior to the students embarking on a DCT PBP. This entailed prescribed readings recommended by the clinical educators, advice on strategies to facilitate more independent practice and a basic clinical knowledge and skills test for students prior to the PBP. Furthermore, students were exposed to discussions around life skills (time management, stress management) to facilitate awareness of potential strategies they can use to cope prior to embarking on DCT. Notwithstanding this, the study revealed the need for more effective preparedness of students for the PBP within DCT platforms.

Beyond student preparedness, the role and scope of the stakeholders involved in the process appeared to not be clearly articulated. The expected role differed to what the participants enacted during DCT. Available time for supervision was a central barrier to effective clinical supervision. Miles and colleagues highlighted potential barriers to effective implementation of DCT, including the shifting roles of the academic supervisors, and the unforeseen workload of clinical educators.^16^ Although documentation outlining expectations of the clinical educator, the academic supervisor and the students were developed in consultation with all stakeholders, this remained an issue.

We postulate that the role confusion highlighted in this study possibly emanated from the impaired communication between the stakeholders of DCT. Clinical educators believed there was inefficient and ineffective communication between the students, clinical educator and academic supervisors. The lack of communication amongst the stakeholders, as well as vague lines of communication, were previously identified as gaps.^13^,^16^,^26^ We are aware that effective communication enables a more positive learning experience for students on the DCT platform,^27^,^28^ hence functioning partnerships between stakeholders are required, to ensure positive learning experiences. This also includes related considerations that have been highlighted by other studies.^13^,^16^,^25^,^29^,^30^ Blose et al^12^, in their study on the roll-out of DCT in physiotherapy at the HEI, supported this view and recommended improved communication between the academic and clinical educators as being an essential part of effective DCT supervision.^12^ The findings of the study, highlighted the need for more frequent engagements and consultation with clinical educators, as well as minimum communication guidelines, to ensure consistent practice.

There were numerous logistical barriers, which negatively influenced the learning environment for students and the experience of supervision for clinical educators. As part of the initial response to mitigate these issues, co-ordinators of the DCT PBP negotiated with clinical educators and the logistic manager for DCT for a more realistic travel schedule, a basic list of consumables for students and a point of contact for clinical educators to discuss logistical issues.

A positive outcome noted in this study was clinical educator's experience of growth in both themselves and the students. This concurs with findings from the two other studies on DCT at the HEI, a study in which physiotherapists reported that their updated clinical knowledge and skills were of value to students,^11^,^13^ and in the study of students and academic supervisors who concurred on the professional development of students in this process.^11^

This study has been useful in highlighting barriers to effective DCT roll out from the clinical educator perspective for OT students, and has also served to illuminate the strengths of such a placement. Further exploration on the strengths of such a programme in the development of students and enhancement of clinical educators practice is required in addition to identifying efficiencies for such placements.

## Conclusion

This study considered the perspective of clinical educators in the pilot roll out of DCT. A clear disconnect was noted in the expectations prior to the implementation of DCT and the actual experiences during DCT supervision. Whilst the experiences of the clinical educators varied, there were similarities around the barriers experienced. Barriers such as poor communication, lack of support from the academic supervisors, lack of resources and time constraints were noted. Enablers included the clinical educators' enthusiasm for sharing their knowledge and the professional growth of both the students and clinical educators. DCT has the potential to be a valuable model of placement and supervision. We however feel that practice placements need to transition towards more rural placements with a PHC focus to facilitate development of graduates who can cope with rural service delivery. Valuable lessons for the co-ordinators of the DCT PBP, were also generated in this study and will aid in enhancing positive student learning experiences on such training platforms. We acknowledge PBP on the DCT platform as essential in promoting this development of contextually-relevant, independent graduates for the public health sector.
